# Testing for extragenital *Neisseria gonorrhoeae* and *Chlamydia trachomatis*: At-home pharyngeal and rectal self-swabs are non-inferior to those completed in healthcare settings

**DOI:** 10.1371/journal.pone.0302785

**Published:** 2024-05-20

**Authors:** Lauren Orser, Vanessa Tran, Patrick O’Byrne, Abigail Kroch, Melissa Bonnetsmueller, Maan Hasso, Alexandra Musten

**Affiliations:** 1 School of Nursing, University of Ottawa, Ottawa, Ontario, Canada; 2 Sexual Health Clinic, Ottawa Public Health, Ottawa, Ontario, Canada; 3 Public Health Ontario, Toronto, Ontario, Canada; 4 Department of Laboratory Medicine and Pathobiology, University of Toronto, Toronto, Ontario, Canada; 5 Ontario HIV Treatment Network, Toronto, Ontario, Canada; 6 Dalla Lana School of Public Health, University of Toronto, Toronto, Ontario, Canada; Hawassa University College of Medicine and Health Sciences, ETHIOPIA

## Abstract

**Introduction:**

The rates of gonorrhea and chlamydia have been increasing in the years preceding the COVID19 pandemic. Because most gonorrhea and chlamydia infections are located in the oropharynx and rectum for men who have sex with men (MSM), and because at-home self-collected swabs for these infections are not licensed by Health Canada or the United States Food and Drug Administration, decreased accessed to in-person care during and since the COVID19 pandemic potentially means missed case findings.

**Objectives:**

To evaluate the performance of at-home self-collected pharyngeal and rectal swabs for gonorrhea and chlamydia nucleic acid amplification testing.

**Methodology:**

All persons who contacted our Sexual Health Clinic and who had a clinical indication to complete oral and/or rectal swabs for gonorrhea and chlamydia were invited to complete at-home swabs in advance of their scheduled appointments. We mailed swabs and instructions to those who consented. Participants brought these swabs to their scheduled in clinic appointments, where we repeated the same swabs. All matching swabs were sent to the laboratory for analysis to determine concordance.

**Results:**

From September 8, 2022 to July 18, 2023, we enrolled 296 eligible participants who provided 1184 swabs. For analysis, cancelled specimens and specimens with invalid results were excluded, leaving 1032 swabs for comparison. We identified 66 STI diagnoses in 47 unique participants. Overall accuracy was high (exceeding 99%), except for rectal chlamydia, which was 96.0%. While the performance of self-swabs for chlamydia was lower compared to gonorrhea, at-home swabs identified six chlamydia infections that were missed by in-clinic collected swabs (two pharyngeal, four rectal). Removing these six cases as “false positives” increased overall accuracy for chlamydia detection to 99.7% (pharyngeal) and 97.8% (rectal).

**Conclusion:**

Self-collected at-home swabs had good performance acceptable for gonorrhea and chlamydia nucleic acid amplification testing.

## Introduction

*Neisseria gonorrhoeae* and *Chlamydia trachomatis* (henceforth gonorrhea and chlamydia) are the most common bacterial sexually transmitted infections (STIs) in Canada [1]. In Ontario, between 2013–2019, the annual incidence rates of gonorrhea infection increased by 128% (from 33.6/100,000 to 76.6/100,000 people), while the annual incidence rates of chlamydia infection increased by 38% (from 256.7/100,000 to 354.3/100,000 people) [2,3]. Decreases were observed during 2020–2021 (gonorrhea: 66.2/100,000; chlamydia: 238.7/100,000 people) [2,3]. These decreases, however, likely occurred due to limited access to testing during the COVID19 pandemic, not due to true reductions in transmission [4].

These ongoing rates of gonorrhea and chlamydia are alarming because these infections have possible sequelae, including, in males, urethritis, testicular and/or epidydimal pain, orchitis, and proctitis, and, in females, pelvic inflammatory disease, chronic pelvic pain, and infertility [1]. Without prompt identification and treatment, *Chlamydia trachomatis* L serovar, i.e., lymphogranuloma venereum (LGV), can cause irreversible damage to lymph tissue [5]. Gonorrhea as well can cause disseminated infection in an estimated 0.5–3% of persons [1]. Lastly, due to the localized inflammation induced by gonorrhea and chlamydia, these infections can increase susceptibility to HIV acquisition, particularly when they occur in the rectum [6,7].

Notably, most gonorrhea and chlamydia infections in men who have sex with men (MSM) are extragenital (i.e., located in the oropharynx and/or rectum) [8]–thus exacerbating HIV vulnerability. Friedman et al. [9] reviewed gonorrhea and chlamydia infections in Ottawa from 2012–2019 and identified that, when using nucleic acid amplification testing (NAAT), 70% of gonorrhea infections and 65% of chlamydia infections were exclusively extragenital. That is, for every three gonorrhea infections that were identified through genital testing, seven infections would have been missed if oral and rectal specimens had not been collected [9].

While research shows that self-swabbing is non-inferior to specimen collection by healthcare professionals [10,11], it is not approved by either Health Canada or the United States Food and Drug Administration for at-home self-collection. At the time of this study, therefore, self-swabbing for extragenital gonorrhea and chlamydia could only occur in healthcare settings in Ontario [12]. Extragenital swabbing for these infections was also not permitted in private laboratories. Due to these restrictions, it is possible that extragenital gonorrhea and chlamydia infections may have been missed in patients seeking testing outside physical clinics (e.g., virtual care, telemedicine, online PrEP clinics). In the context of (1) increased rates of gonorrhea and chlamydia, yet decreased access to clinics for testing during the COVID19 pandemic, and (2) the possible negative effects of these infections related to pathophysiology and risk of HIV acquisition, we evaluated the performance of at-home self-collected pharyngeal and rectal swabs for gonorrhea and chlamydia NAAT.

## Methods

This study took place at the Sexual Health Clinic in Ottawa between September 2022 and July 2023. During this time, eligible persons who contacted the Sexual Health Clinic by phone for an appointment were invited to participate in the study at their upcoming clinic visit. Full study information was given verbally over the phone to obtain initial consent, which was documented and witnessed on a participant recruitment form. To be eligible, participants had to be ≥16 years old and not taking antibiotics, and they had to meet the Public Health Ontario recommendations for extragenital testing (Table 1) [12]. Those who consented were mailed, in advance of their upcoming appointment, a test kit package, which included Roche Cobas® swabs for pharyngeal and/or rectal specimen collection, and instruction sheets with text and image-based instructions plus links to online videos about how to perform the swabs [13], and labels to mark which swab contained the pharyngeal or rectal specimen. Participants were instructed to complete their specimens at home and bring these swabs to their upcoming scheduled in-person clinical visit, where they reviewed and signed a research consent form, the clinician collected the at-home swabs, and the matching swabs were repeated in clinic by either the participant or the clinician (based on patient preference). Per the storage specifications of the swab sample kit, specimens were valid for 12 months from collection if stored at room temperature (between 2–30°C) [12,14]. To ensure accuracy of the specimens, we used 30 days as a maximum restriction between at-home and in clinic specimen collection. Participants who did not bring their at-home swabs or who did not collect these swabs correctly (e.g., used the incorrect swab, did not label specimens) were excluded from the study.

**Table 1:** Recommendations for rectal and/or pharyngeal testing [12].

Sexually active persons who belong to groups with elevated STI prevalence, including: • Gay, bisexual, trans, and other men who have sex with men • Persons engaging in sex work or persons who had sexual contact with sex worker(s) • Persons with a sexual exposure to a lab confirmed case of chlamydia and/or gonorrhea • Persons with symptoms of a rectal and/or pharyngeal infection

All swabs were sent to Public Health Ontario’s laboratory for testing, where they underwent analysis using the cobas® CT/NG assay on the cobas® 8800 system (Roche Diagnostics, Branchburg, NJ, USA). The cobas® CT/NG assay is a qualitative real-time PCR that detects gonorrhea and chlamydia DNA simultaneously. The laboratory testing protocol mandated that positive gonorrhea results were confirmed using the PivNG assay V2 on the Roche cobas® omni utility channel. Possible test results were positive, negative, inconclusive, and invalid. Reasons for invalid results included interfering substances inhibiting the real-time PCR reaction or suboptimal content in the specimen. Clinically, we treated any positive result as a true positive (irrespective of discordant test results), provided treatment and did partner follow-up and management. We also considered inconclusive results as positive. Our rationale was that an inconclusive result indicated that at least one repeat test was positive and, clinically, it would merit follow up. We recommended repeat testing for any inconclusive or invalid test results, with these being done by clinicians. In the event of concordant negative results, we recommended repeat screening per guidelines (e.g., every 3 months for men who have sex with men) [1,12]. Manufacturer reported performance characteristics for the Roche Cobas® assay are listed in Table 2 [14]. Rectal specimens from males that tested positive for chlamydia were sent to the National Microbiology Laboratory (NML; Winnipeg, Manitoba, Canada) for LGV testing.

**Table 2:** Sensitivity and specificity of extragenital NAATs [14].

	Gonorrhea		Chlamydia	
	*Sensitivity*	*Specificity*	*Sensitivity*	*Specificity*
*Pharyngeal*	100% (96.2–100%)	98.9% (98.4–99.2%)	100% (87.9–100%)	99.8% (99.6–99.9%)
*Rectal*	99.0% (94.6–99.8%)	99.3% (98.9–99.6%)	95.1% (90.2–97.6%)	99.2% (98.8–99.5%)

### Data collection

We used an Excel spreadsheet to track study data. Participants were logged according to their clinical chart number, which was used to review files following their visits. For eligible participants (those who brought their specimens to the visit and had collected them correctly), we noted timing of at-home collected swabs, if new sexual contact(s) had occurred, and test results received from the laboratory (positive, negative, inconclusive, invalid). For ineligible participants, we noted reason for ineligibility (e.g., did not attend visit, did not bring swabs, etc.), but did not record test results. For participants with a confirmed positive result, we recorded which swab was positive (essentially, the site of infection), and if the results were concordant for all swabs. We also noted LGV results for any participant with a confirmed rectal chlamydia infection.

### Sample size

We determined that 50 positive test results must be obtained from clinic swabs with concordant at-home swabs to determine within p≤0.01 that at-home swabs were non-inferior to in-clinic swabs within 88–100%. Using the positivity rate of the Sexual Health Clinic (8% for chlamydia, 4% for gonorrhea), we estimated we would need 400 tests to be conducted to obtain an adequate number of positive results to compare sensitivity of at-home and in-clinic swabs.

### Data analysis

We analyzed the data descriptively to report on means and frequencies. The swabs were analyzed by infection (gonorrhea, chlamydia) and site (pharyngeal, rectal).

We tabulated sensitivity and specificity, and negative and positive predictive values for the tests by infection and site, using in-clinic collected swabs as the “gold standard”. Sensitivity was calculated as the total number of concordant positive test results for each infection by site divided by the total number of identified infections per site using the in-clinic collected swabs (i.e., true positives/(true positives and false negatives)). Specificity was calculated in the same way using negative results (i.e.; true negatives/(true negatives and false positives)). The positivity predictive value was calculated as the total number of concordant positive test results (true positives) divided by the total number of true positives and false positives. Negative predictive value was calculated in the same way using negative results (i.e.; true negatives/(true negatives and false negatives)).

To determine if individuals performing at-home swabs collected a similar amount of bacterial DNA versus the quantity collected by in-clinic swabs, we compared the cycle threshold (Ct) values of paired positive swabs. Although Ct values are not a quantitative measurement of bacterial DNA, these values are inversely proportional to the amount of DNA in a sample, such that a higher Ct value indicates that lower amounts of DNA are present. We completed a simple linear regression of the Ct values between positive pairs by creating a scatter plot of Ct values with in-clinic swabs on the x-axis and at-home swabs on the y-axis in Excel. A line-of-best fit was created to determine the correlation coefficient (r^2^) and slope.

### Ethics and funding

The Research Ethics Board at the University of Ottawa approved this project (H-02-22-7856). Individuals provided verbal consent for participation at the time of recruitment which was documented and witnessed on a participant recruitment form. Written consent for study participation was also obtained during the clinical visit prior to submitting test swabs. Minors (under the age of 16 years) were not eligible to participate in this study; therefore, we did not require consent from parents or guardians. Funding for this study was provided by the Ontario HIV Treatment Network (EFP-2020-DC1).

## Results

From September 8, 2022 to July 18, 2023, we offered enrollment in the STI validation study to 810 persons who contacted the Sexual Health Clinic for an appointment; 477 consented to collect at-home swabs and 38% (n = 181/477) were ineligible at the time of appointment. The most common reasons for ineligibility were that persons did not bring their at-home swabs to their appointment (n = 112), they did not attend their appointment (n = 59), or they made a specimen collection error (n = 7), including using the flocked swab (instead of woven) or putting both the flocked and woven swabs into the specimen collection tube. Three additional samples were cancelled by the laboratory due to incorrect labelling. The study thus included 296 eligible participants who provided 1184 swabs. For analysis, cancelled specimens and specimens with invalid results were excluded, leaving 1032 swabs for comparison. Among the 296 eligible participants, the average age was 34 years (minimum: 19; maximum: 77). Most participants (n = 280) identified as male; an additional 5% (n = 16) identified as female. The average time between at-home collected specimens and in clinic collected specimens was 10.3 hours.

### Positive test results

For positive test results, we identified 66 STI diagnoses in 47 unique participants. The average time between at-home and in clinic specimen collection was 10.6 hours, with collection time not available for 3 participants. The majority of persons with a positive result identified as cis-male (n = 44/47 or 94%) and were an average age of 37 years old (minimum: 20 years old; maximum: 65 years old). For risk factors, 96% (n = 45/47) were MSM and 4% (n = 2/47) reported sex work. A full 79% (n = 37/47) of these participants had a negative HIV screening result at the time of their visit; half of whom reported using PrEP daily for HIV prevention. The other 10 participants with a positive result were noted to be living with HIV, all of whom were engaged in HIV treatment and care at the time of their sexual health visit. In terms of reason for testing, nearly 50% (n = 23/47) of persons with a positive result had presented for asymptomatic STI screening, 34% (n = 16/47) had a sexual contact who was recently diagnosed with an STI (with or without symptoms), and 17% (n = 8/47) had presented with symptoms suggestive of an STI.

### Sensitivity and specificity

Overall, the accuracy of the at-home swabs was ≥99%, except for the rectal swabs for chlamydia testing, which was 96.0%. The sensitivity and specificity of the at-home swabs for gonorrhea testing were 100% and >99%, respectively (Table 3). The sensitivities of the at-home swabs for chlamydia testing were lower, at 83.3% for pharyngeal samples and 82.1% for rectal samples (Table 3). The specificities of the at-home swabs for chlamydia testing were 99.3% for pharyngeal samples and 98.0% for rectal samples (Table 3).

**Table 3:** Performance characteristics of at-home self-collected swabs for gonorrhea and chlamydia NAAT.

Target	Site	Overall Accuracy % [95% CI], (n)	Adjusted Overall Accuracy* % [95% CI], (n)	Sensitivity % [95% CI], (n)	Specificity % [95% CI], (n)	Adjusted Specificity* % [95% CI], (n)	Positive Predictive Value % [95% CI], (n)	Adjusted Positive Predictive Value* % [95% CI], (n)	Negative Predictive Value % [95% CI], (n)
*Neisseria gonorrhoeae*	Pharyngeal	99.7 [98.1–99.99], (288/289)	n/a	100.0 [80.5–100.0], (17/17)	99.6 [98.0–99.99], (271/272)	n/a	94.4 [70.6–99.2], (17/18)	n/a	100.0 [98.7–100.0], (271/271)
	Rectal	100.0 [98.4–100.0], (227/227)	n/a	100.0 [71.5–100.0], (11/11)	100.0 [98.3–100.0], (216/216)	n/a	100.0 [71.5–100.0], (11/11)	n/a	100.0 [98.3–100.0], (216/216)
*Chlamydia trachomatis*	Pharyngeal	99.0 [97.0–99.8], (286/289)	99.7 [98.1–99.99], 286/287)	83.3 [35.9–99.6], (5/6)	99.3 [97.5–99.9], (281/283)	100.0 [98.7–100.0], (281/281)	71.4 [37.5–91.2], (5/7)	100.0 [47.8–100.0], (5/5)	99.6 [97.1–99.9], (281/282)
	Rectal	96.0 [92.6–98.2], (218/227)	97.8 [94.9–99.3], (218/223)	82.1 [63.1–93.9], (23/28)	98.0 [94.9–99.5], (195/199)	100.0 [98.1–100.0], (195/195)	85.2 [68.2–93.9], (23/27)	100.0 [85.2–100.0], (23/23)	97.5 [94.6–98.9], (195/200)

*Adjusted following investigation of discordant results identified false positives as true positives.

Overall, 13 discordant results were identified, all of which were among participants who completed self-collection for both at-home and in clinic specimens. More discordant results for chlamydia were observed (n = 12) than for gonorrhea (n = 1). An investigation of discordant results is summarized in Table 4. For the chlamydia discordant results, the Ct value was generally high (>35), except for two, which had Ct values around 30. In 42% (n = 5/12) of these cases, the participant was also identified as having chlamydia at the other anatomical site.

**Table 4:** Investigation of discordant results.

Discordant Results		Investigation
Chlamydia Pharyngeal	At-home negative & in-clinic positive (n = 1)	1—Ct of clinic-collected swab was 37.79. Repeat testing was Ct 37.24. All other specimens from patient were negative.
	At-home positive & in-clinic negative (n = 2)	1—Ct 30.46. Patient was positive for rectal chlamydia, so this is likely a true positive (i.e., missed by clinic pharyngeal swab) 2 –Ct 36.83, repeat testing with Ct 36.33. Patient was positive for rectal-chlamydia so this is likely a true positive (i.e., missed by clinic pharyngeal swab)
Chlamydia Rectal	At-home negative & in-clinic positive (n = 5)	1 –Ct of clinic swab was 33.01 (confirmed by NML) 2 –Ct of clinic swab was 30.91 (confirmed by NML) 3 –Ct of clinic swab 36.15 (confirmed by NML) 4 –negative/inconclusive pair. Ct of clinic swab was 41.52. Patient positive for pharyngeal chlamydia. 5 –negative/inconclusive pair. Ct of clinic swab was 39.93 (repeat testing was negative). All other specimens from the patient were negative.
	At-home positive & in-clinic negative (n = 4)	1 –Ct 37.86. Confirmed by NML so likely a true positive (i.e., missed by clinic swab) 2 –Ct 35.66, confirmed by NML so likely true positive 3 –Ct 37.03, confirmed by NML so likely true positive. All other specimens from patient were negative. 4 –Ct 35.05, confirmed by NML so likely true positive. All other specimens from the patient were negative.
Gonorrhea Pharyngeal	At-home positive & in-clinic negative (n = 1)	1 –Inconclusive (Ct 39.17)/negative pair. All other specimens were negative. Patient was positive for pharyngeal gonorrhea two weeks prior. The at-home swab may be a false positive or detecting residual DNA.

Two participants had positive pharyngeal swabs for chlamydia from at-home collection but negative swabs from in-clinic collection. These two participants were also positive for rectal chlamydia, suggesting that the at-home pharyngeal self-swabs were true positives that were missed by the in-clinic collected swabs. Another four participants had positive rectal swabs for chlamydia from at-home collection but negative swabs from in-clinic collection. These four at-home specimens were confirmed by the NML; therefore, they are true positive results that were missed by the in-clinic-collected swabs. Overall, the at-home-collected swabs identified infections missed by in-clinic collected swabs in six cases of chlamydia (two pharyngeal, four rectal).

Six participants had negative swabs for chlamydia from at-home collection but positive swabs from in-clinic collection (one pharyngeal and five rectal swabs). Two were negative/inconclusive pairs and two had Ct values >35 for the in-clinic swab and two had Ct values of 30–33 for the in-clinic swabs. Three of these specimens were confirmed by the NML, demonstrating that these were missed by the at-home swabs. One participant had pharyngeal chlamydia, suggesting the at-home swab missed this infection. The remaining two did not have any other identified infections but had Ct values >37.5.

Four participants with rectal chlamydia were identified as having LGV by the NML. The at-home swab identified 100% of these infections (n = 4/4), whereas in-clinic specimen collection missed one case (75% detection, n = 3/4).

One participant had a positive pharyngeal swab for gonorrhea from at-home collection but a negative swab from in-clinic collection. This result was an inconclusive/negative pair with very high Ct value. This patient had been diagnosed and treated for gonorrhea 2 weeks prior.

Taking the chlamydia results that were positive at-home and negative in-clinic to be true positive results, there were six discordant results, of which most (5/6) were among specimens with Ct values >35. Removing these six cases from analysis increased overall accuracy for chlamydia detection to 99.7% (pharyngeal) and 97.8% (rectal), and specificity to 100% (Table 3).

### Cycle threshold (Ct) comparison

**Fig 1:**
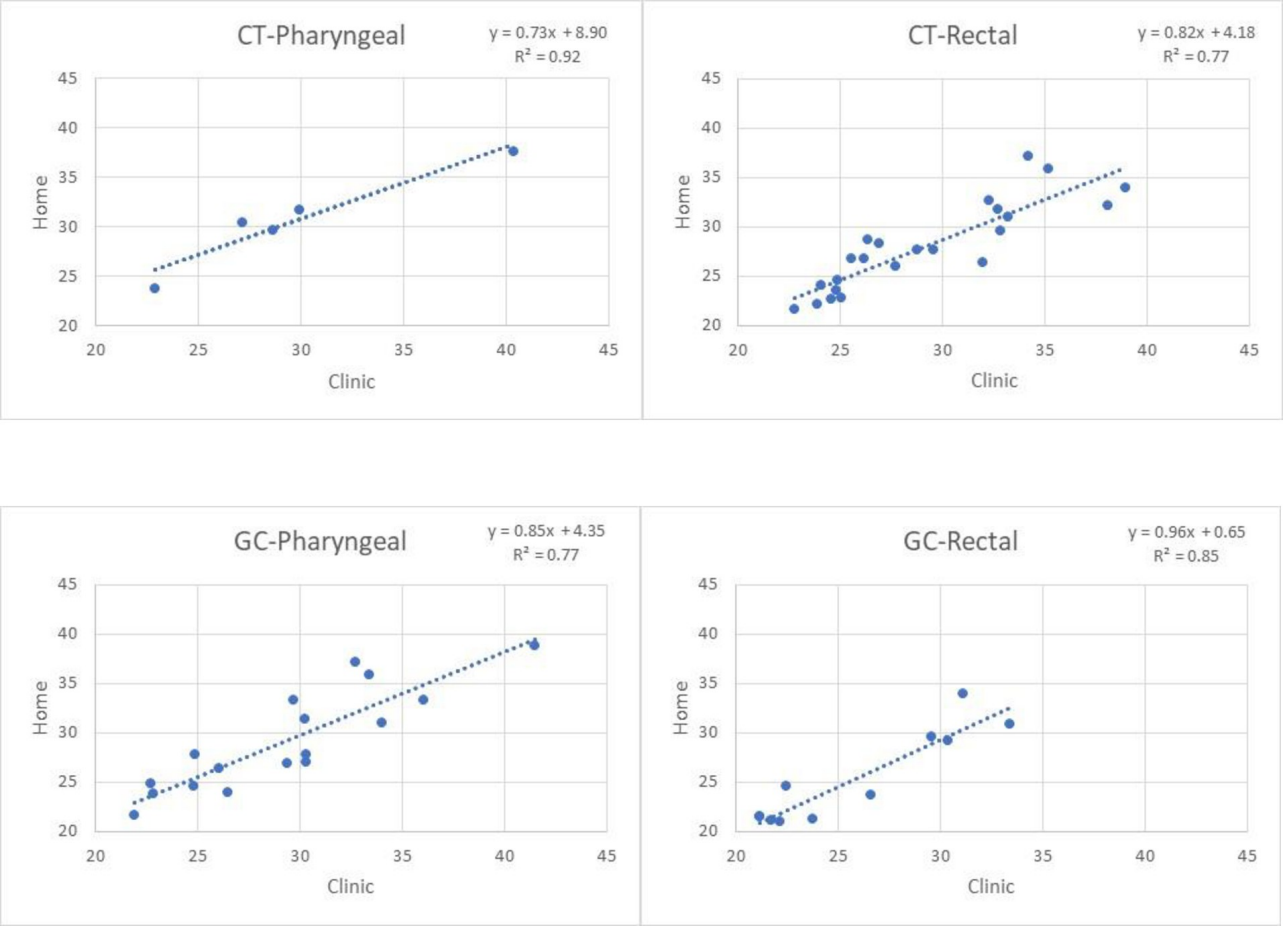
Linear regression analysis of Ct values for positive pairs.

The Ct values were comparable between the at-home collected swabs compared to the in-clinic collected swabs. Linear regression analyses of the Ct values between positive pairs are depicted below by site (Fig 1) Overall, 86% (48/56) of swab pairs had less than three Ct values difference between the at-home-collected swabs compared to the in-clinic-collected swabs. Of the 56 positive pairs, 57% (32/56) of the at-home self-collected swabs had lower Ct values compared to the in-clinic-collected swabs. Taken together, this suggests that specimen collection is not compromised by at-home self-swabbing.

## Discussion

In this study, we compared the performance of at-home versus in-clinic self-collected swabs to detect pharyngeal and rectal gonorrhea and chlamydia. Overall accuracy was high (exceeding 99%), except for rectal chlamydia, which was 96.0%. While the performance of self-swabs for chlamydia was lower compared to gonorrhea, at-home swabs identified six chlamydia infections that were missed by in-clinic collected swabs (two pharyngeal, four rectal). Our investigation of these six discordant results identified that they were all in fact true positives, which increased overall accuracy for chlamydia detection to 99.7% (pharyngeal) and 97.8% (rectal) and specificity to 100%. Of the 1032 swabs tested only 13 discordant results were obtained, most of which were with specimens with a high Ct value (Ct>35). Specimens with high Ct values are subject to more variability due to the low amount of bacterial DNA that is present in the swab being tested. Notably, 92% (12/13) of the discordant results were obtained with chlamydia testing, and this is likely due to the fact that *C*. *trachomatis* is an obligate intracellular bacterium with alternating extracellular, infectious elementary body and intracellular, non-infectious reticulate body [15,16], which means that swabbing for this organism requires cellular (not just exudate) collection that necessitates longer and more direct contact between the swab material and the affected mucosal membranes. These results raise a few points for discussion.

First, our results suggest that at-home specimen collection for pharyngeal and rectal gonorrhea and chlamydia had similar detection rates and performance compared to in-clinic specimen collection. It was also interesting that, while the Ct values for over 80% of these samples were within three Ct values of each other, over 50% of at-home chlamydia swabs had lower Ct values compared to Ct values from in-clinic specimens; this suggests more robust swabbing at-home, compared to in-clinic. These findings align with a growing body of research, which has consistently identified that at-home swabs for these two infections is equivalent–if not superior–to specimen collection that occurs in clinical settings [10,11]. This is a reassuring finding in the context of (1) increased and increasing rates of gonorrhea and chlamydia, (2) a burden of these infections among MSM that is often located in the pharynx and rectum, (3) decreased access to testing for gonorrhea and chlamydia during and since the COVID19 pandemic, and (4) an inability heretofore for patients to complete at-home swabs in our and many other jurisdictions [1–3,9,17]. It is, moreover, important to expand access to testing for rectal gonorrhea and chlamydia, as these infections at this anatomical site are known to increase biologic vulnerability to HIV acquisition among MSM [6,7]. At-home specimen collection is thus a viable option both to increase such access and to decrease demand on screening in brick-and-mortar STI clinics.

Second, our results highlight that while at-home testing for extragenital gonorrhea and chlamydia functioned for many, it did not work for everyone. Approximately 38% of persons who consented to our study became ineligible once they attended their clinic visit. Most often (94% of the time), ineligibility occurred because participants had not brought their swabs to the clinic visit; indeed, only 6% (n = 7) were ineligible because they made overt errors in the at-home specimen collection process. While this finding corresponds with the literature, which identifies that most people can complete self-swabbing with good accuracy [10,11,18], it also highlights a key item for those who wish to implement at-home swabbing for gonorrhea and chlamydia: consider making swabs available at laboratory drop-off locations, where patients can complete their swabs on-site when they provide urine and/or blood specimens for other testing. While our participants were still able to be tested (because they had presented to a clinic), if these patients had been completing at-home swabs which they deposited at laboratories for processing, some persons might have received incomplete testing (i.e., extragenital infections might have been missed if these persons forgot to bring swabs to the laboratory for testing). This could lead to delayed diagnosis, sequelae, and onward transmission because many gonorrhea and chlamydia infections are extragenital in MSM [9]. It can also leave other persons at elevated risk for HIV due to an undiagnosed rectal infection [6,7]. If such on-site specimen collection is not permitted at laboratories, to ensure good quality patient care, clinicians should monitor their laboratory results and encourage completion of the pharyngeal and rectal swabs among those who did not take them to their laboratory visits.

Third, our results showed that, even among participants whose specimens were processed at the laboratory, some still had not completed the swabbing correctly, although this applied equally to at-home and in-clinic specimen collection. In addition to highlighting the need for patients to receive clear instructions about how to complete swabbing, particularly for rectal self-collection (including the need to insert the swab 3-5cm into the anal canal and to rotate it for 5–10 seconds while pressing the swab against the rectal mucosa), our findings suggest that patients should be encouraged to complete both pharyngeal and rectal swabs at every testing episode. Over 40% of chlamydia infections that were missed on one swab were identified at the other anatomical site, which ensured prompt identification and treatment of infection for these participants. This recommendation to complete comprehensive testing aligns with literature which has identified similarly high rates of rectal infections among MSM who deny receptive anal sex ranging between 20–35% [19–21]. Another lesson from this study is that clinicians should consider retesting patients if they obtain negative test results but have a high clinical suspicion of infection (e.g., if the patient is symptomatic or is a contact of someone diagnosed with chlamydia or gonorrhea). This repeat testing could then identify an infection that was potentially missed through at-home self-swabbing.

### Limitations

These results must be interpreted considering certain limitations. First, the study occurred in one city in Canada, which has a STI testing clinic and population exceeding 1 million people. Different performance outcomes may have been identified if clinicians were not as well versed in providing patients with instructions on how to use these swabs. Conversely, higher uptake of at-home self-swabbing may be observed in areas without an STI clinic if self-swabbing were the only access to testing and care. Second, our study was restricted to persons who fulfilled the laboratory-approved list for extragenital gonorrhea and chlamydia testing (Table 1). Again, different performance outcomes could arise if at-home pharyngeal and rectal self-swabbing were offered to members of other groups who were seeking STI testing. Finally, participants in this study conducted regular STI testing, and many were already accustomed to self-swabbing. Different results may have arisen in persons who were completing STI testing for the first time.

## Conclusions

In an effort to expand access to gonorrhea and chlamydia testing of the pharynx and rectum, we evaluated the performance of at-home self-swabs to those completed in-clinic. We identified that, overall, self-collected at-home swabs had good performance acceptable for gonorrhea and chlamydia NAAT. Given the lower sensitivity for chlamydia as self-collected at-home swabs, we recommend that both rectal and pharyngeal swabs should be collected if at-home self-swabbing is done. In addition, clinicians should consider retesting when there is high clinical suspicion of infection. Despite these shortcomings, it appears that at-home self-swabbing to test for gonorrhea and chlamydia NAAT is a viable option that could expand access to testing. Considering the increasing rates of these infections, the challenges in accessing in-person care, and the risks these infections put persons at, this new strategy is timely and needed.
